# An Improved Azimuth Angle Estimation Method with a Single Acoustic Vector Sensor Based on an Active Sonar Detection System

**DOI:** 10.3390/s17020412

**Published:** 2017-02-20

**Authors:** Anbang Zhao, Lin Ma, Xuefei Ma, Juan Hui

**Affiliations:** 1 College of Underwater Acoustic Engineering, Harbin Engineering University, Harbin 150001, China; zhaoanbang@hrbeu.edu.cn (A.Z.); malin@hrbeu.edu.cn (L.M.); huijuan@hrbeu.edu.cn (J.H.); 2 Science and Technology Underwater Acoustic Laboratory, Harbin Engineering University, Harbin 150001, China

**Keywords:** active sonar detection system, single AVS, matched filtering, azimuth angle estimation, complex acoustic intensity measurement, time-domain processing

## Abstract

In this paper, an improved azimuth angle estimation method with a single acoustic vector sensor (AVS) is proposed based on matched filtering theory. The proposed method is mainly applied in an active sonar detection system. According to the conventional passive method based on complex acoustic intensity measurement, the mathematical and physical model of this proposed method is described in detail. The computer simulation and lake experiments results indicate that this method can realize the azimuth angle estimation with high precision by using only a single AVS. Compared with the conventional method, the proposed method achieves better estimation performance. Moreover, the proposed method does not require complex operations in frequency-domain and achieves computational complexity reduction.

## 1. Introduction

Acoustic vector sensors (AVS) can measure both the acoustic pressure and acoustic particle velocity at a signal point in space, and therefore can capture more sound field information than conventional acoustic pressure sensors [1,2]. AVS and AVS arrays have been long used for target direction of arrival (DOA) estimation and localization [3,4,5]. Recently, AVS has been implemented in many other engineering applications, such as underwater acoustic communication [6], port and waterway security [7], pipeline protection [8], geo-acoustic inversion problems [9], and sound field analysis [10]. Consequently, research on the AVS signal processing methods is of great engineering and practical significance.

Compared with processing only based on acoustic pressure, processing based on pressure and particle velocity can achieve better detection effectiveness and higher estimation precision [11]. With the continuous development of underwater acoustic technology, sonar array systems and the underwater acoustic array signal processing have attracted increasing research attention. In [1], the measurement model of an AVS array for estimation issues is presented in detail. Both conventional and minimum variance beamforming methods for DOA estimation by using AVS arrays are introduced in [12]. The wideband minimum variance distortionless response (MVDR) beamforming method by an AVS linear array is presented in [13], which is based on coherent signal subspace preprocessing, to solve the problem of DOA estimation for wideband coherent acoustic sources. In [14], one blind coherent two-dimensional DOA estimation algorithm is proposed for arbitrarily spaced AVS arrays that subject to unknown locations. One novel sensor array model is introduced in [15], which is based on higher-dimensional second-order statistics of received data at the sensors.

Although the AVS array processing can provide better detection and estimation performance [16], the larger scale represents the main restriction in its application area. Therefore, in some engineering applications, single-transmit single-receive (SISO) sonar systems, such as sonobuoys and other small-scale detection equipment, are widely used their layout convenience characteristics, high stealthiness, low complexity, etc. The DOA estimation of the underwater target can be achieved by using a single AVS [2,3,4,5]. Therefore, research on the single vector sensor signal processing is important for practical engineering applications. The DOA estimation based on average acoustic intensity processing is proposed in [17]. In [18], the authors proposed a beamforming approach to DOA estimation based on a single AVS. Another new azimuth angle estimation method through complex acoustic intensity measurement is put forward in [19]. The simulation results indicated that the method can effectively detect the target and estimate its azimuth angle in a coherent interference background. A novel method based on complex acoustic intensity weighted statistics and another method aiming to detect a target that radiating line-spectra are proposed in [20]. However, in the multi-targets situation, the method based on the average acoustic intensity processing can only measure the direction of the synthetic acoustic intensity and it cannot distinguish the azimuth angles of multiple targets. The line-spectra azimuth angle estimation and the weighted statistics azimuth angle estimation based on complex acoustic intensity measurement both have excellent performance. Moreover, the approach based on complex acoustic intensity weighted statistics can suppress broadband coherent interference [20].

In underwater acoustic target detection, active-echo detection is the only effective way to detect an acoustically passive or a quiet target [21]. In the existing literature, all researches referred to above on single vector sensor DOA estimation are based on passive detection systems. In some cases, these approaches can be extensively applied in an active detection system. However, they are not so well suited for the active detection. Consequently, in order to meet practical engineering application demands in active sonar detection system, the authors propose one improved azimuth angle estimation method based on taking advantage of the matched filtering processing gain of single AVS.

The major contributions of this paper can be listed as follows:

1. This paper focuses on the signal processing on the active detection system. Based on practical engineering applications, it presents an improved azimuth angle estimation method by using a single AVS as the receiver.
2. In comparison with the conventional method, the theoretical description is deduced in detail. Computer modeling and simulation are done to verify the performance of the proposed method. Moreover, a series of open water experiments are carried out, which are very challenging.
3. Real measurement data processing results and practical performance analysis of both the conventional method and the proposed method are provided in this paper.
4. The low computational complexity method proposed in this paper provides a new and valuable insight into the active sonar detection system. In addition, AVS can be not only used to estimate azimuth angle of the target, but also applied to suppress direct acoustic signal interferences. Although the latter is not discussed in this paper, it is of great significance for active sonar detection systems.

The rest part of this paper is organized as follows: Section 2 demonstrates the theoretical measurement model for azimuth angle estimation based on a single AVS. Section 3 describes the proposed estimation method in detail. In addition, the conventional method based on complex acoustic intensity measurement is introduced in this part as a comparison. Computer simulation results are presented in Section 4. Section 5 consists of lake experiments, data processing results, and estimation performance discussion. A summary of the proposed method is provided in Section 6.

## 2. The Measurement Model

**Figure 1:**
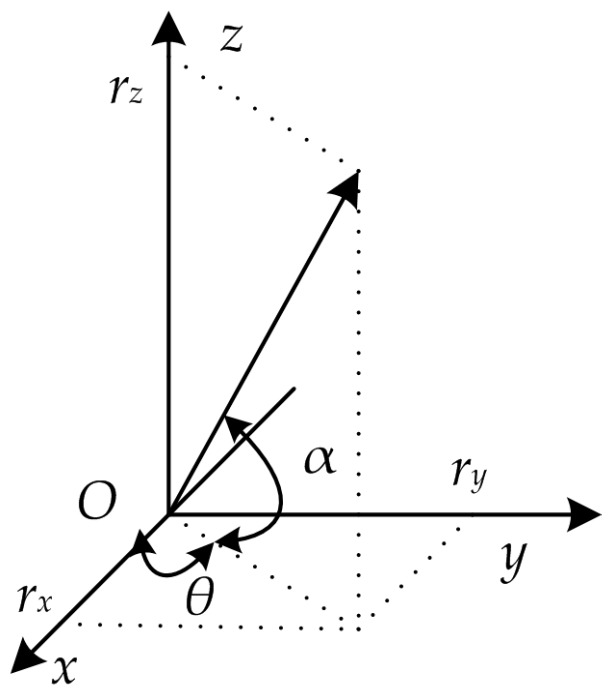
Projection of a wave vector in the Cartesian coordinate system.

Throughout this paper, acoustic waves are assumed to be propagating in a quiescent, homogeneous and isotropic fluid. For the impinging signals, we always assume that they are plane waves, i.e., the hydrophone is located in the far field of the acoustic sources. According to the classical acoustic theory, the plane wave acoustic pressure at a frequency is expressed as [22]:

(1) $$p(r,t)=p_{0}e^{j(k^{T}\cdot r-\omega t)}$$

where *ω* is the angular frequency, *k* is the wavenumber vector, *r* is space vector. As shown in Figure 1, the signal impinges upon the hydrophone from an azimuth angle *θ* and with an elevation angle *α*.

Any point in a three-dimensional (3-D) space can be represented by a vector **r** = [*r_x_*, *r_y_*, *r_z_*]*^T^*, where *r_x_*, *r_y_*, *r_z_* are the coordinates of the point in the Cartesian coordinate system. Equation (1) can be expressed as:

(2) $$p(r,t)=p_{0}\exp[j(kr_{x}\cos\theta \cos\alpha +kr_{y}\sin\theta \cos\alpha +kr_{z}\sin\alpha -\omega t)]$$

Then, we substitute Equation (2) into Euler’s Equation (also named the Equation of Motion) [2,3,4]:

(3) $$\frac{\partial v}{\partial t}+\frac{1}{\rho }\nabla p=0$$

where *ρ* denotes the medium density and we have:

(4) $$v(r,t)=\frac{p(r,t)}{\rho c}(\cos\theta \cos\alpha \vec{\xi }+\sin\theta \cos\alpha \vec{\eta }+\sin\alpha \vec{\zeta })$$

where *ζ*, *η*, *ξ* denotes the unit vector of *x*, *y* and *z* axis. Only the azimuth angle *θ* is considered in this paper, and the elevation angle *α* is neglected. In following measurement models, the acoustic pressure and acoustic particle velocity depend on time only. The argument **r** is omitted from these variables.

The acoustic pressure component and two orthogonal components of particle velocity in the *x*-*O*-*y* plane could be obtained by using AVS. The co-point output mathematical expression of the measurement model can be finally given as:

(5) $$\{\begin{array}{c} p(t)=p(t)\\ v_{x}(t)=\frac{1}{\rho c}p(t)\cos\theta \\ v_{y}(t)=\frac{1}{\rho c}p(t)\sin\theta \end{array}$$

The velocity direction characteristics of AVS are frequency-independent. Therefore, the directivity pattern of AVS is also frequency-independent. Consequently, even for acoustic signals in very low frequency, a small-sized AVS still has dipole directivity pattern. It is the physical basis for a single AVS to measure the direction of the arriving signals.

## 3. Propose Improved Method

In [20], the authors proposed two approaches to estimate the azimuth angle by a single AVS based on complex acoustic intensity measurement. One is the bar graph method and the other one is the weighted bar graph method. Both methods are based on complex acoustic intensity measurements, but they differ in their statistical weighted value. All the method referred to above can be applied in both passive and active sonar detection systems. The method proposed in this paper is mainly for active detection systems, however, for better illustration, the conventional azimuth estimation algorithm based on complex acoustic intensity measurement is introduced in advance.

### 3.1. The Conventional Method

**Figure 2:**
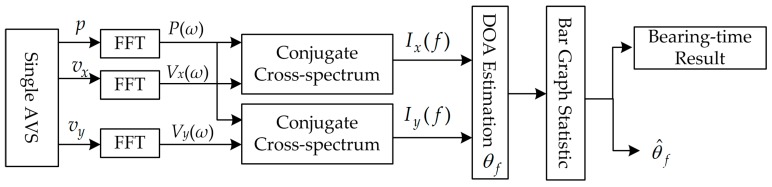
Principle chart of bar graph approach based on complex acoustic intensity measurements.

The processing flowchart of the conventional method is shown in Figure 2 [2,20]. Based on the conjugate cross-spectrum, the azimuth angle is calculated corresponding to each frequency. Then a weighted statistics bar graph is used to obtain the azimuth estimation curve.

Applying a Fast Fourier Transform (FFT) to *p*(*t*) and *v_i_*(*t*) (*i* = *x*, *y*) with respect to the variable *t*, we can obtain the frequency spectrum *P*(*ω*) and *V_i_*(*ω*) (*i* = *x*, *y*). The acoustic pressure and velocity cross-spectrum can be expressed as [2]:

(6) $$S_{pv_{i}}(\omega )=P(\omega )\cdot V_{i}^{*}(\omega )\;(i=x,y)$$

where ‘*’ denotes the complex conjugate operation.

We employ a sliding window averaging operation in the time-domain. The average periodgram output can then be expressed as:

(7) $$<S_{pv_{i}}(\omega )>=<P(\omega )\cdot V_{i}^{*}(\omega )>\;(i=x,y)$$

where ‘<·>’ denotes sliding- average periodgram operation.

In the underwater acoustic channel, the acoustic Ohm’s law is approximately satisfied. Therefore, the acoustic pressure signal has the same phase as the velocity signal. According to the basic properties of the Fourier transform, the energy of two signals in the same phase is concentrated on the real component of the cross-spectrum. Consequently, the energy of the detected target signal concentrates on the real component of the cross-spectrum. The imaginary component of the output only contains the energy of interference and noise. We have:

(8) IRi=Re{<P(ω)⋅Vi*(ω)>} (i=x,y)

where, ‘Re’ denotes the operation that getting the real component:

(9) θ(ω)=arctanIRyIRx=arctanRe{<P(ω)Vy*(ω)>}Re{<P(ω)Vx*(ω)>}

First, we calculate the azimuth angle of each frequency based on the acoustic pressure and velocity conjugate cross-spectrum. Then we determine the probability density statistics based on the azimuth angle calculation results of all frequency points, to get the azimuth estimation curve at a certain moment. The corresponding maximum value on the curve is the target azimuth angle estimation result.

The conventional bar graph statistics is expressed as:

(10) k=[θ(f)×180/π]

(11) δ(k)=δ(k)+1

where *k* is referred to as angle (in degrees), ‘[]’ denotes getting integer operation. *δ* is an array, which stores the frequency of each angle in [0, 360]. The initial value of *δ* is zero. Note that the statistics weighted value is one in each frequency.

Obviously, the method referred to above is not so reasonable. Therefore, the improved method is substituting the weighted value with the energy of cross-spectrum vary in different frequency point. Namely:

(12) δ(k)=δ(k)+W

where *W* denotes the statistic weighted value of cross-spectrum based on complex acoustic intensity measurement.

All the methods described above are from passive sonar detection systems. Certainly, they could also be applied in active sonar detection systems. However, the biggest advantage of the active sonar detection system is that the detection signal is known in advance and the algorithm processing gain can be obtained from the a priori information of the detected signal. Therefore, if the algorithm of the passive processing system is simply used, the partial processing gain is lost. Consequently, a novel active azimuth angle estimation method is proposed based on the conventional passive one.

### 3.2. The Improved Method

Under the assumption of Gaussian white background noise, matched filtering is the best output according to the maximum Signal-to-Noise Ratio (SNR) criterion [23]. The system impulse response function of matched filter can be expressed as:

(13) h(τ)=s(T−τ)

In the physical sense, the impulse response of the matched filtering system is the time reversal and shift of the original signal. The response function in frequency-domain can be obtained by Fourier transform:

(14) $$H(\omega )=S^{*}(\omega )e^{-j\omega T}$$

If the observation time *T* is set to 0, then:

(15) h(τ)=s(−τ)

In the frequency-domain:

(16) $$H(\omega )=S^{*}(\omega )$$

Consequently, the physical impulse response function is just the time reversal of the original signal. The impulse response function in frequency-domain is the conjugate spectrum of the original signal. According to Fourier transform characteristics, convolution in the time-domain corresponds to a multiplication in the frequency-domain [24]:

(17) $$s(t)*s(-t)\Leftrightarrow S(\omega )\cdot S^{*}(\omega )$$

Therefore, the azimuth angle estimation can be realized by matched filtering in the time-domain in a similar processing method. The corresponding equation is deduced as:

(18) $$\theta =\arctan\frac{\left[p(t)*s(t)][v_{y}*s(t)\right]}{\left[p(t)*s(t)][v_{x}*s(t)\right]}$$

where ‘∗’ denotes matched filtering operation, *s*(*t*) denotes the original transmitting signal.

Note that in Equation (18), the component ’*p*(*t*) ∗ *s*(*t*)’ appears in both the denominator and numerator, but it cannot be simplified. The operation ‘arctan’ results range is [−*π*/2, *π*/2], but the target locates in [0, 2*π*]. To avoid spatial aliasing, target quadrants is determined based on the sign of denominator and numerator. Consequently, the sign of the component ’*p*(*t*) ∗ *s*(*t*)’ cannot be omitted.

**Figure 3:**
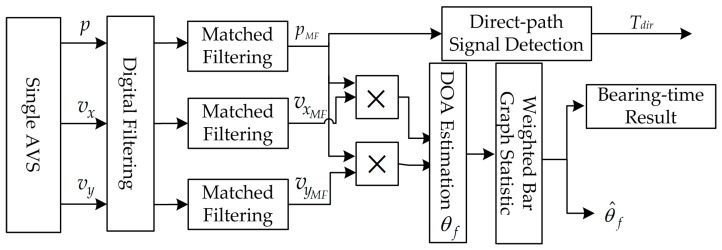
Principle chart of azimuth angle estimation based on matched filtering.

The proposed improved algorithm processing flowchart is shown in Figure 3.

The signal measured by the AVS not only contains the desired signal but also contains noise and interference. The digital filter is designed to effectively denoise the received signal. Then, matched filtering operation is applied to the three-channel signal, respectively. Then, we calculate the azimuth angle corresponding to each point by Equation (18).

The same as the conventional method, the probability density statistics based on the azimuth angle calculation results is introduced to get the azimuth estimation curve. Bar graph estimation is a statistical approach. The statistics weighted value ‘W’ of this improved method is the energy in each corresponding point. Consequently, the curve maximum position corresponds to the estimated value of which is the target position.

By comparing Equations (9) and (18), we can easily find that in the complex acoustic intensity measurement, the cross-spectrum contains only the self-information of the passively received signal. Thus, it makes no use of the advantage that the active detection signal priori information. Under ideal circumstances, the processing gain of matched filtering is:

*G* = 10log*BT* (19)

where *B* is the frequency band, *T* is the length of the detection signal.

Matched filtering is always widely applied in practical underwater acoustic detection and estimation. The proposed method aims to improve azimuth angle estimation performance in active sonar systems mainly by making use of the matched filtering processing gain. The performances of the conventional method and the proposed method are discussed and compared in the following section.

## 4. Simulation

In order to verify the effectiveness of the proposed algorithm, computer modeling and simulation are carried out. The modeling and simulation parameters are shown in Table 1.

### 4.1. The Detection Signal

**Figure 4:**
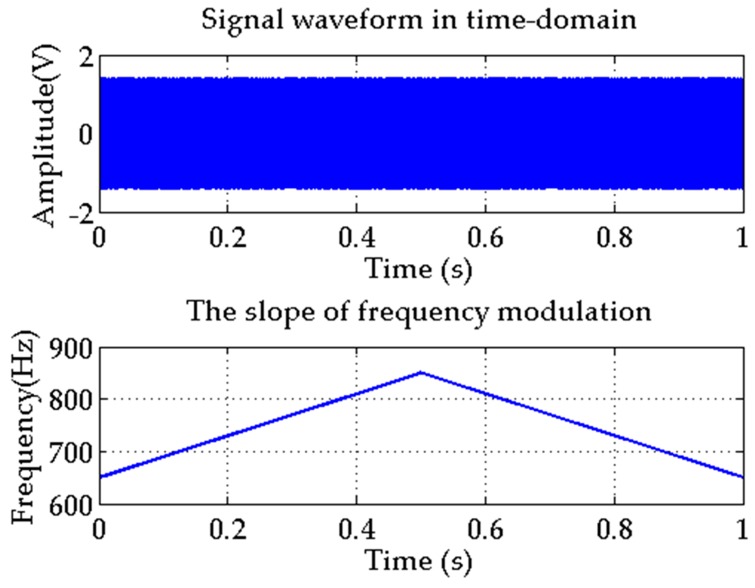
Detection signal waveform in time-domain and the slope of frequency modulation.

The active detection signal applied in simulations and lake experiments is a 650–850 Hz symmetrical chirp signal [26]. The time-domain waveform and signal frequency changing trend are shown in Figure 4.

This signal is characterized by:

(20) s(t)=s(−t)

According to the matched filtering theory, its response function is the signal itself. Therefore, there is no need to employ a time reversal operation. Moreover, we note that matched filtering operation applied in Equation (18) can be substituted by convolution [27]. Under this signal condition, the operation can be further simplified. In addition, to a certain extent, the ability of this form signal to resist channel distortion is much better than the conventional asymmetric chirp signal.

### 4.2. Underwater Acoustic Channel Simulations

**Figure 5:**
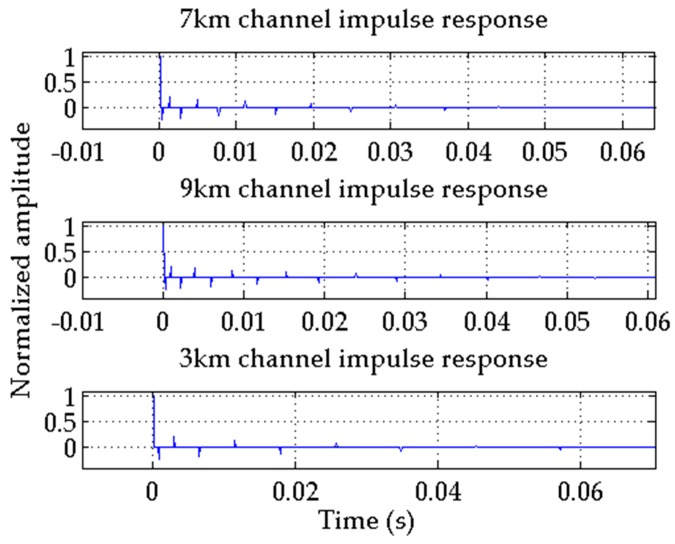
Channel impulse responses.

The three-parameter-ray channel impulse response simulation model is established to calculate the channel impulse response function with different parameters [28]. The results are shown in Figure 5.

### 4.3. Processing Results

**Figure 6:**
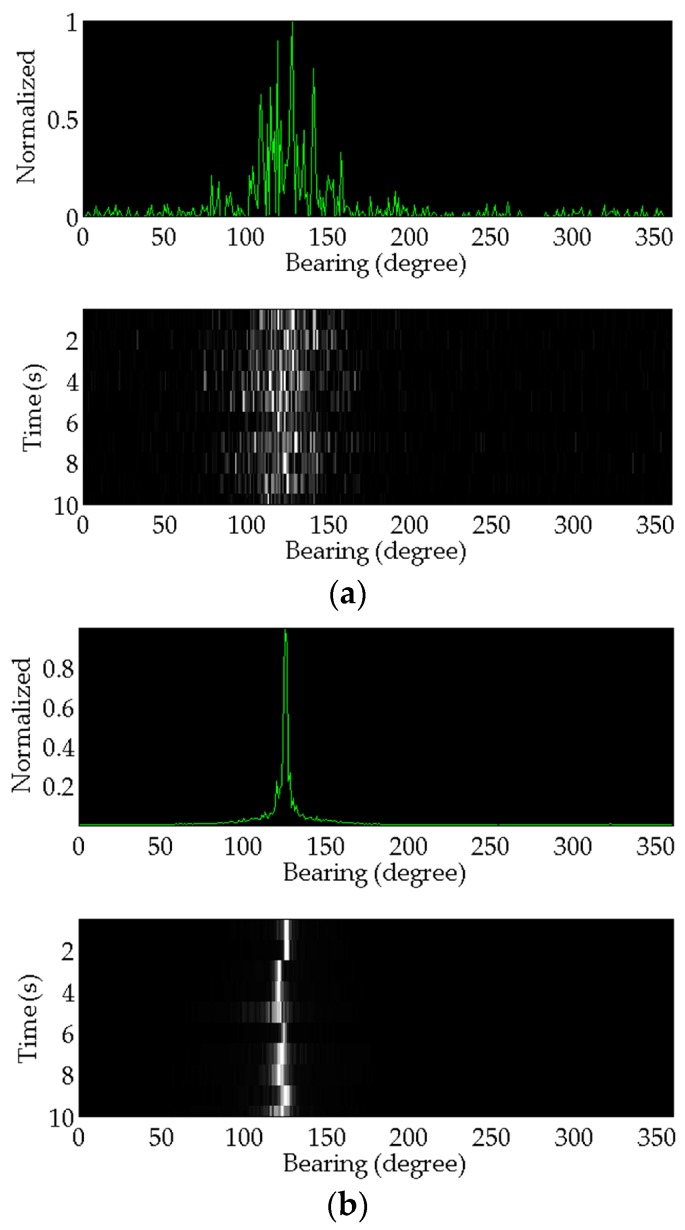
The processing results: (**a**) The result of the conventional method; (**b**) The result of the proposed method.

The proposed method modeling and simulation results are compared and analyzed with that of the conventional method. These target azimuth angle estimation results are shown in Figure 6. The top subfigure shows the most recent azimuth estimation results. Bearing-time results are simultaneously shown in the subfigure below.

**Figure 7:**
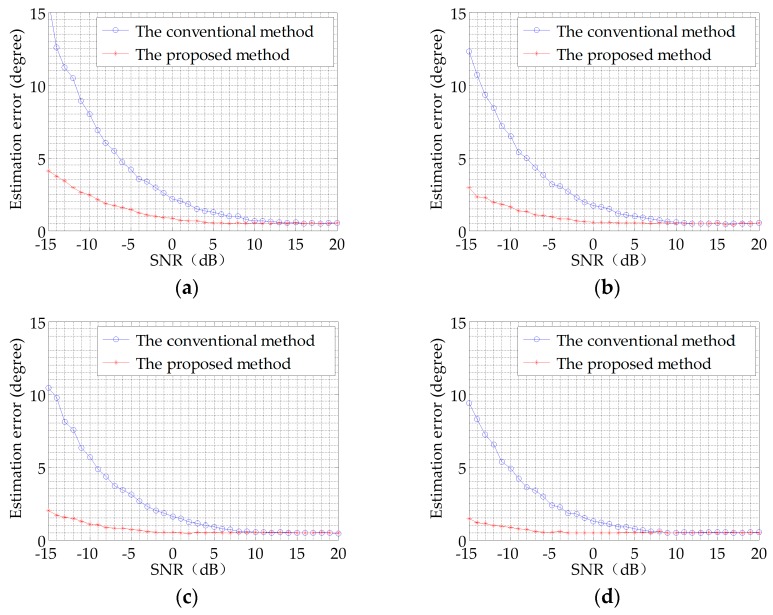
Comparison of DOA estimation error between two methods: (**a**) the signal length is 0.2 s; (**b**) the signal length is 0.5 s; (**c**) the signal length is 1 s; (**d**) the signal length is 2 s.

Comparing the results, the performance of the proposed method is much better than that of the conventional one. The azimuth angle estimation is much more accurate. To illustrate the advantage of the proposed method, Monte Carlo simulation method with 500 trials is applied to verify the statistical error of the two different algorithms. Under the Gaussian noise background, the SNR is varying from −15 dB to 20 dB. Four symmetrical chirp signals different in length are simulated, respectively. These results are shown in Figure 7.

Comparing the results, we note that the azimuth angle estimation error based on the matched filtering is much less than that based on the complex acoustic intensity measurement. It indicates that the azimuth angle estimation performance of the proposed method is much better than that of the conventional method. For the longer signal, we obtained the smaller error of the azimuth angle estimation. Consistent with Equation (19), the longer the signal, the greater the processing gains we achieve.

## 5. Lake Experiments and Results

**Figure 8:**
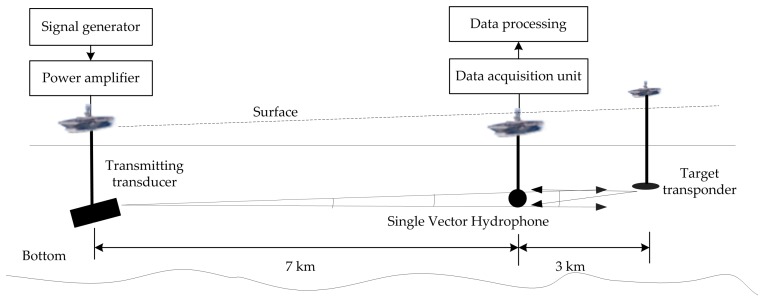
The schematic diagram of the open water experimental layout.

In order to further verify the practical application performance of the proposed algorithm, the research group carried out open lake water experiments in the Danjiangkou reservoir from April to June 2016. The experimental layout in open water is shown in Figure 8. The three ships used in the experiments are identified as follows:

- Acoustic Source Ship, carrying high power transmitting sound source;
- Target Transponder Ship, equipped with target analog transponder;
- Receiver Ship, carrying vector hydrophones and other related electronic devices.

As the equipment conditions were limited, the three boats applied in the experiments were anchored. The AVS and electronic equipment cabin are suspended through a cable. It is a non-rigid connection. Under the water, the AVS rotates with the water flow, so the device must be equipped with a magnetic compass to track the attitude of the underwater vector hydrophone. According to consulted related information, the angle between the North geographic pole and the North magnetic pole at the experimental site is 4°. The bottom of the lake is muddy and sandy in general.

**Figure 9:**
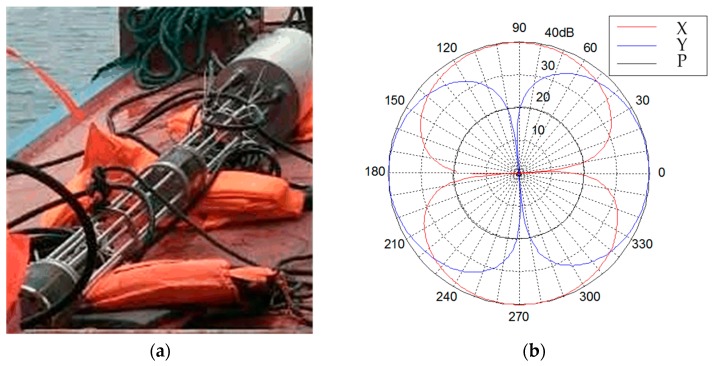
(**a**) The AVS and electronic equipment cabin used in the experiments; (**b**) The AVS directivity patterns (*@*630 Hz).

Figure 9a shows the AVS mounted at the measurement structure. The AVS applied in lake experiments is a 2-D accelerometer structure sensor and its directivity patterns (@630 Hz) are shown in Figure 9b. Its pressure sensitivity pattern is omnidirectional. The acceleration sensitivity on each axis offers a lateral rejection ratio of 35 dB or more against the other orthogonal axis.

**Figure 10:**
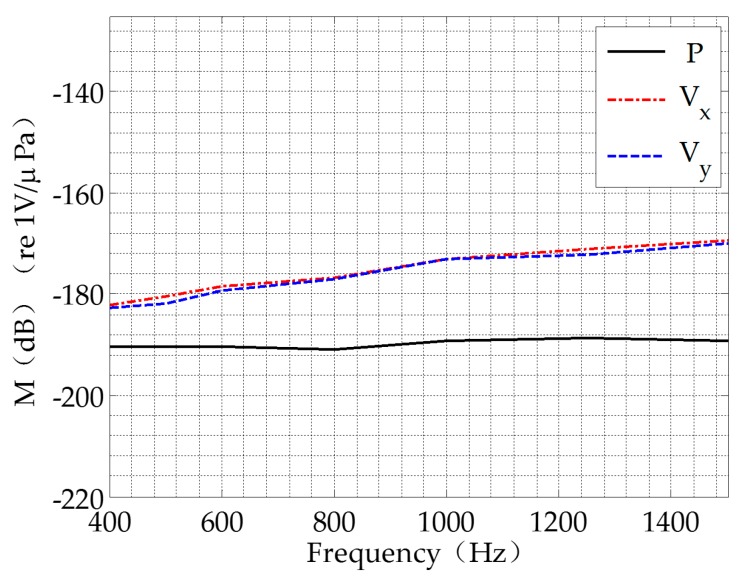
The AVS sensitivity between 400 and 1500 Hz.

The AVS provides a maximum receiving sensitivity of −188.8 dB re μPa between 400 and 1500 Hz. The results are shown in Figure 10. In these experiments, the data acquisition unit is used to acquire data from the AVS. Six channels are used for the two acoustic vector hydrophones. The total sampling rate is 250,000.

**Figure 11:**
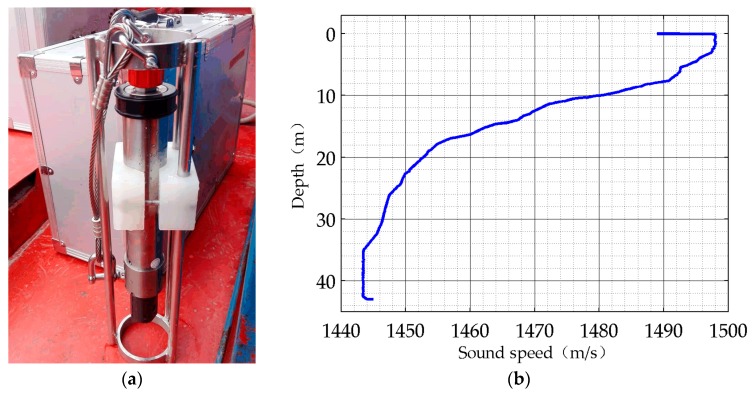
(**a**) The CTD; (**b**) The sound speed profile.

The sound speed profile is measured by the CTD on the receiver ship at the beginning of the experiments. The CTD used in the experiments and the measured sound speed profile are shown in Figure 11.

It can be clearly seen from the measured sound speed profile that there is a serious negative sound speed gradient layer. In this layer, the sound wave propagation is refracted downward. Therefore, it works against acoustic signal transmission. Below the depth of 35 m, an equal velocity profile layer appears. According to underwater acoustic theory, this layer is suitable for acoustic signal transmission. However, considering the bottom of the experiments site is an undulating area, we make a compromise and place the equipment down to 25 m under the water. The negative sound speed gradient in this layer is much smaller than that of the upper layer. Meanwhile, it is easy to avoid the terrain undulation influence. The GPS coordinates of the three ships during the lake experiments, and the actual field-measured environmental parameters are listed in Table 2.

According to the GPS coordinates, the actual distance and angle are calculated. The actual distance between the receiver ship and the acoustic source ship is 7067.9 m, and the actual angle between the connecting line and the geographic North Pole is 26.9°. The actual distance between the receiver ship and the target transponder ship is 3059.2 m. The actual angle between the connecting line and the geographic North Pole is 191.8°. The actual distance between the target ship and the acoustic source ship is 10,053.9 m.

In the lake experiments, the actual sound source level (SL) of the transmitting transducer is 194.6 dB re μPa. Propagating in the underwater acoustic channel, the signal is received by the single vector hydrophone and the target transponder, respectively. The target transponder is triggered by the received signal. And then, the target transponder forwards the received signal to simulate the real target echo. The target strength is set as 10 dB re μPa in the experiments.

**Figure 12:**
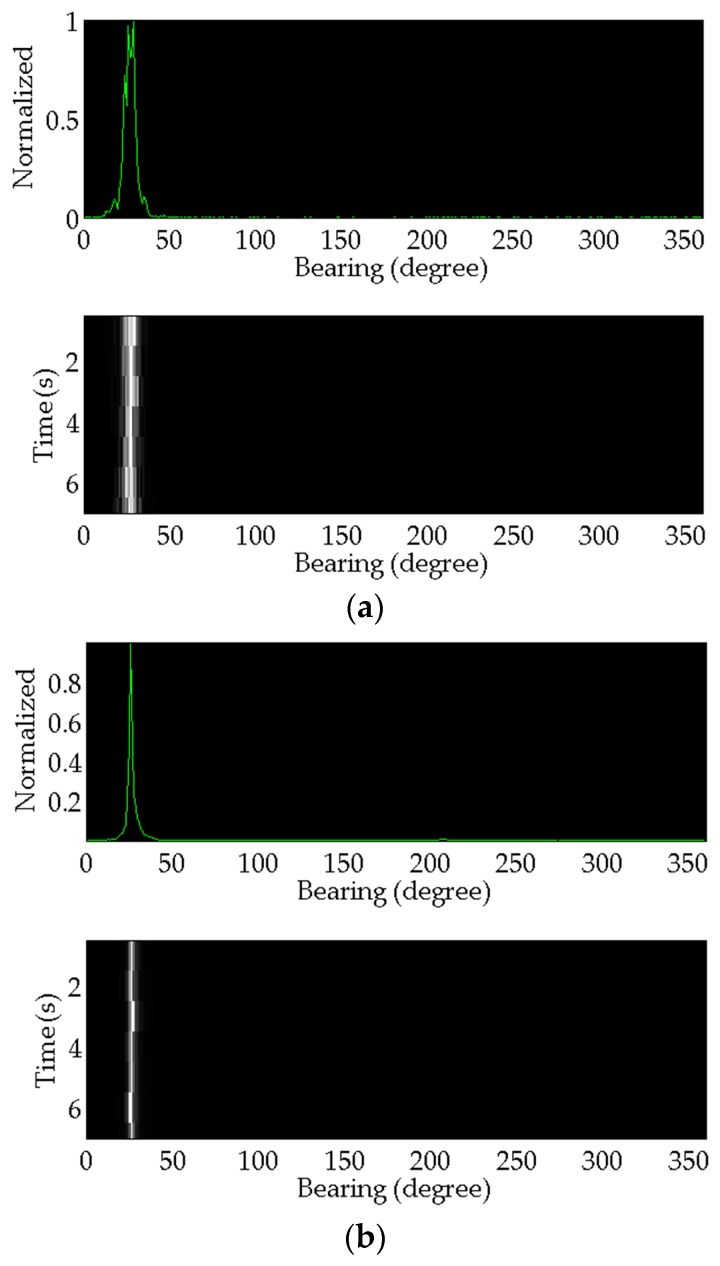
The azimuth angle estimation results of the acoustic source ship. (**a**) The result of the conventional method; (**b**) The result of the proposed method.

**Figure 13:**
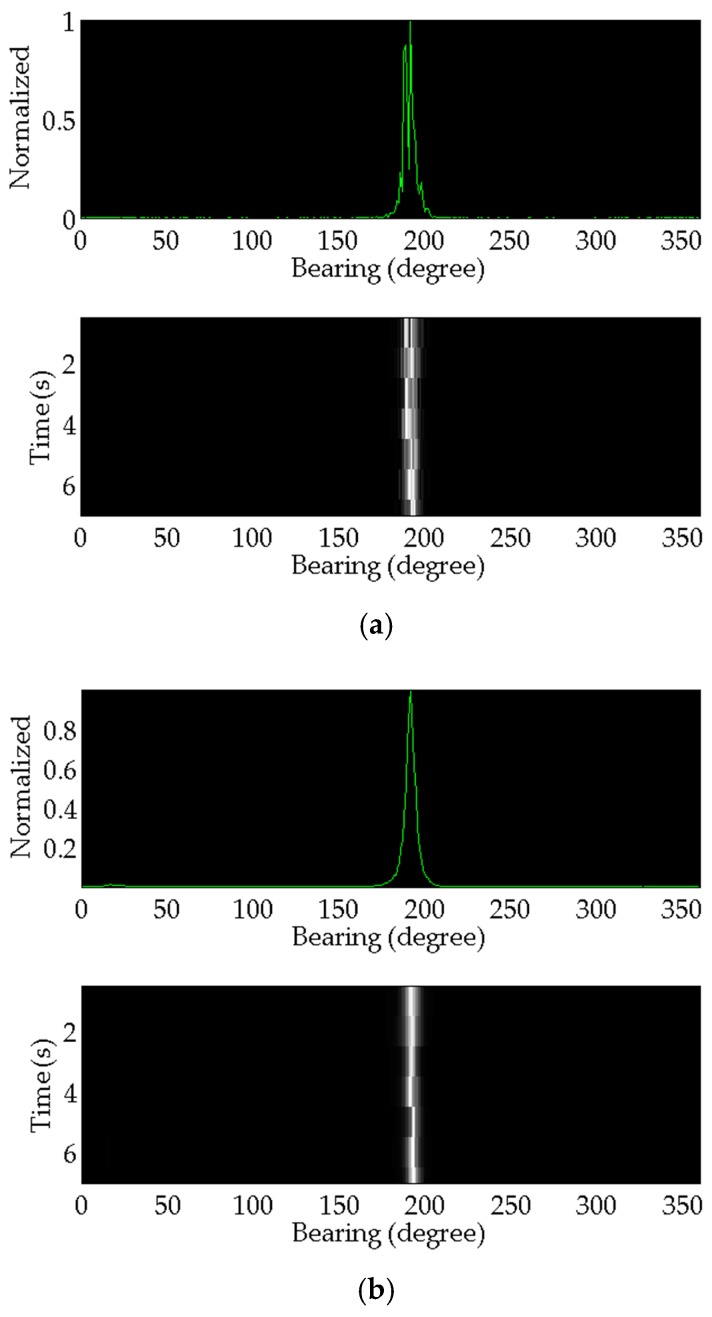
The azimuth angle estimation results of the target transponder ship. (**a**) The result of the conventional method; (**b**) Th result of the proposed method.

The signal received by acoustic vector sensor is acquired by thendata acquisition unit. Meanwhile, the data is uploaded to the data processing computer. Then, the two methods introduced above are applied to estimate the target azimuth angle. The processing results of the lake experiments data are shown below. Figure 12 shows the estimation results of the acoustic source ship. Figure 13 shows the target transponder ship results.

The azimuth angle estimation results of seven times lake experiment data are listed in detail in Table 3. The lake experiment data processing results are indicated by numbers in the parameter column.

Comparing the results of the two methods, the target azimuth angle estimation results variance processed by the conventional method is larger than that of the proposed method. This indicates the conventional method’s stability performance is much worse. In respect of the relative error, the accuracy of the proposed method is higher than that of the conventional one. Consequently, the comprehensive performance of the proposed azimuth angle estimation method in this paper is much better than that of the conventional one.

## 6. Conclusions

In this paper, an improved method based on a single AVS is proposed for azimuth angle estimation in active detection systems. The physical and mathematical principles are well studied and deduced. Meanwhile, computer modeling and simulation are applied to demonstrate the proposed algorithm’s reasonableness. Based on lake experiments and data processing results, the effectiveness of the algorithm is verified. Compared with the traditional method, the proposed one has a higher angular resolution, smaller error, and higher stability. All of this shows the superiority of the proposed method. In practical engineering applications, the proposed algorithm is implemented in the time-domain, without Fourier transformation. It does not require complex operations and achieves computational complexity reduction. However, it remains to be determined whether the proposed method can achieve good performance or not in a multi-target environment.

## Figures and Tables

**Table 1:** Modeling and simulation parameters.

Parameters	Value
baseline length	7000 (m)
L_tr_^1^	3000 (m)
L_tt_^2^	9000 (m)
azimuth angle	123 (°)
water depth	40 (m)
D_eu_^3^	20 (m)
NL ^4^	70 (dB)
transmission loss [25]	15l g (R) ^5^

^1^ L_tr_: distance between target and receiver; ^2^ L_tt_: distance between transducer and target; ^3^ D_eu_: depth of equipment in underwater; ^4^ NL: underwater environment noise level; ^5^ R: transmission distance.

**Table 2:** Experiments geographical environment parameters.

Parameters	Acoustic Source Ship	Receiver Ship	Target Transponder Ship
GPS coordinates	32°45.683′ N 111°33.807′ E	32°42.272′ N 111°31.762′ E	32°40.652′ N 111°31.361′ E
water depth (m)	38	43	46
D_eu_ (m)	20	25	25

**Table 3:** Analysis of the target azimuth angle estimation result.

Parameters	The Conventional Method (°)	The Improved Method (°)
1	191.5	192.5
2	190.9	191.9
3	191.5	192.5
4	189.4	191.4
5	189.1	192.1
6	192.2	192.2
7	191.9	191.9
Mean value	190.9	192.1
Variance	1.48	0.13
Estimation error	0.9	0.3
